# Refractory Ventricular Tachycardia and Seizures With Lacosamide Overdose

**DOI:** 10.7759/cureus.29547

**Published:** 2022-09-25

**Authors:** Chris Marcellino, Chase C Ransom, Eelco F Wijdicks

**Affiliations:** 1 Department of Neurology, Mayo Clinic, Rochester, USA; 2 Department of Anesthesia, Mayo Clinic, Rochester, USA; 3 Department of Neurologic Surgery, Mayo Clinic Health System, Eau Claire, USA; 4 Department of Neurologic Surgery, Mayo Clinic, Rochester, USA

**Keywords:** eeg, stellate ganglion block, seizure, overdose, cardiac arrest, ventricular tachycardia, lacosamide

## Abstract

We describe a 60-year-old female patient who suffered an apparently intentional overdose of lacosamide and who developed status epilepticus secondary to its toxicity, complicated by refractory ventricular arrhythmia necessitating advanced cardiac life support and percutaneous stellate ganglion blockade. Extracorporeal membrane oxygenation was considered, and arterial and venous small-bore sheaths were placed in order to allow for extracorporeal cardiopulmonary resuscitation if cardiac arrest recurred, but they were not ultimately used. She suffered an embolic left middle cerebral artery stroke but otherwise recovered from the episode. This eventful clinical course highlights the dangers of lacosamide in high doses.

## Introduction

Lacosamide, an anticonvulsant compound approved in 2008 for the adjunctive treatment of partial-onset seizures and neuropathic pain, can in overdose, paradoxically, cause both seizures and terminal cardiac arrhythmias [1-3].

## Case presentation

**Figure 1:**
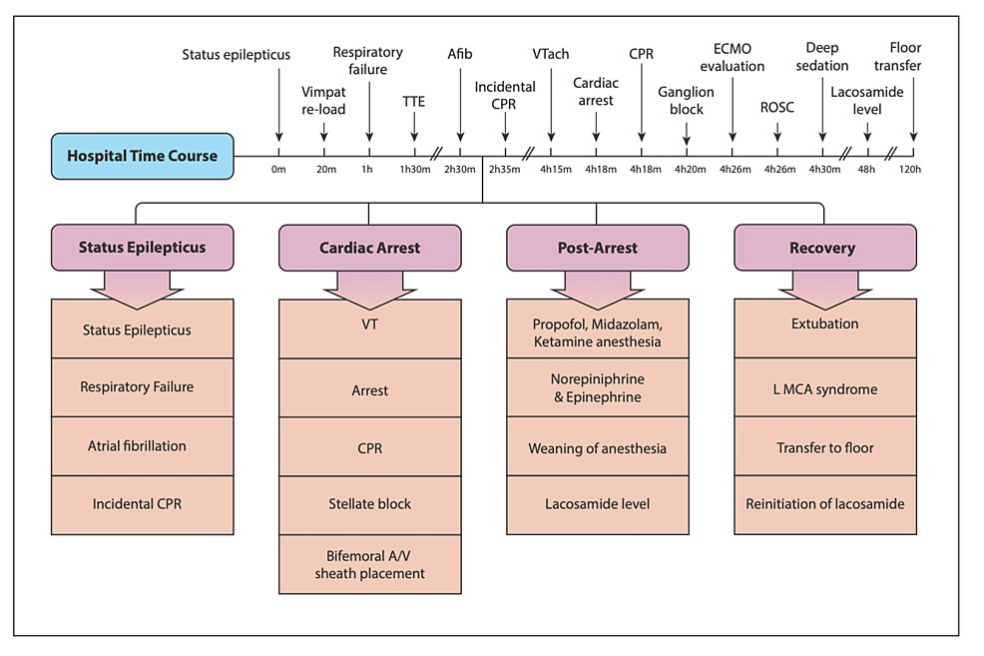
Time course This timeline summarizes the patient’s hospital course starting at status epilepticus, which developed shortly after admission as evidenced by the deterioration of her examination and EEG findings, through her recovery and transfer to general care. CPR - cardiopulmonary resuscitation, VT - ventricular tachycardia, A/V - arterial and venous, L MCA - left middle cerebral artery, TTE - transthoracic echocardiogram, Afib - atrial fibrillation, VTach - ventricular tachycardia heartbeat, ECMO - extracorporeal membrane oxygenation, ROSC - return of spontaneous circulation

A 60-year-old female, with a past medical history of non-lesional and reportedly well-controlled bifrontal epilepsy maintained on levetiracetam and lacosamide (1500 and 200 mg total daily dosing, respectively) with only rare breakthrough seizures (less than annually), chronic obstructive pulmonary disease, hypertension, coronary arterial disease, history of stress-induced cardiomyopathy, anxiety, depression, and history of alcohol use disorder, presented to the ED after a breakthrough generalized seizure. She was noted to have a productive cough and subsequent focal seizures, which progressed to status epilepticus (see time course in Figure 1). She was treated with intravenous (IV) benzodiazepines and given additional IV doses of home medications (levetiracetam 2000 mg and lacosamide 200 mg) to abort her seizures as it was suspected that she had not taken her medications on the day of presentation. She developed acute hypoxic respiratory failure and obtundation, which was thought to be related to the escalation of antiseizure therapy, and subsequently required intubation and intensive care unit (ICU) admission. Prior to ICU admission, she was in normal sinus rhythm and reportedly without significant telemetry abnormalities. The patient was connected to continuous video electroencephalography (EEG). At times, she was noted to be in atrial fibrillation. Over the next two hours, she had multiple sequential brief generalized seizures, after which she developed a narrow complex rapid tachycardia with a rate of approximately 260-280 bpm. She developed hypotension requiring multiple vasopressors and fluid resuscitation; a transthoracic echocardiogram showed mild enlargement of the right ventricle with associated depressed systolic function (in the setting of positive pressure ventilation) with preserved left ventricular ejection fraction. Cardiac enzymes and cerebrospinal fluid profile were unremarkable. 

**Figure 2:**
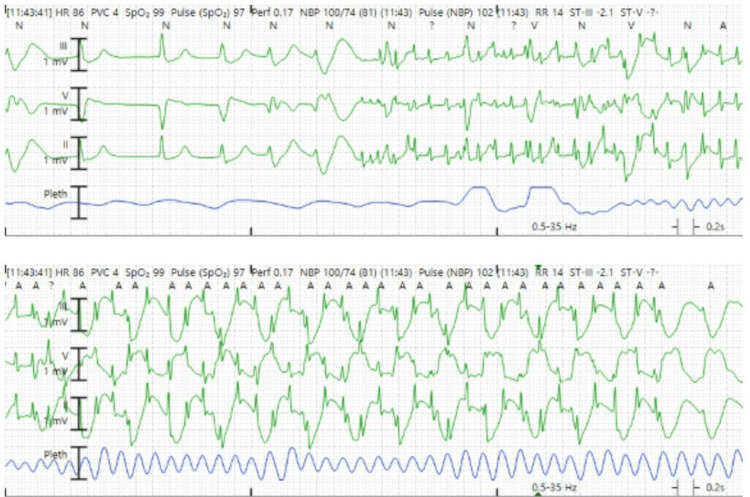
Cardiac arrest Wide complex rapid ventricular tachycardia, which rapidly leads to cardiac arrest. The ventricular tachycardia cycle length was approximately 400 ms with a rate of approximately 260-280 bpm.

**Figure 3:**
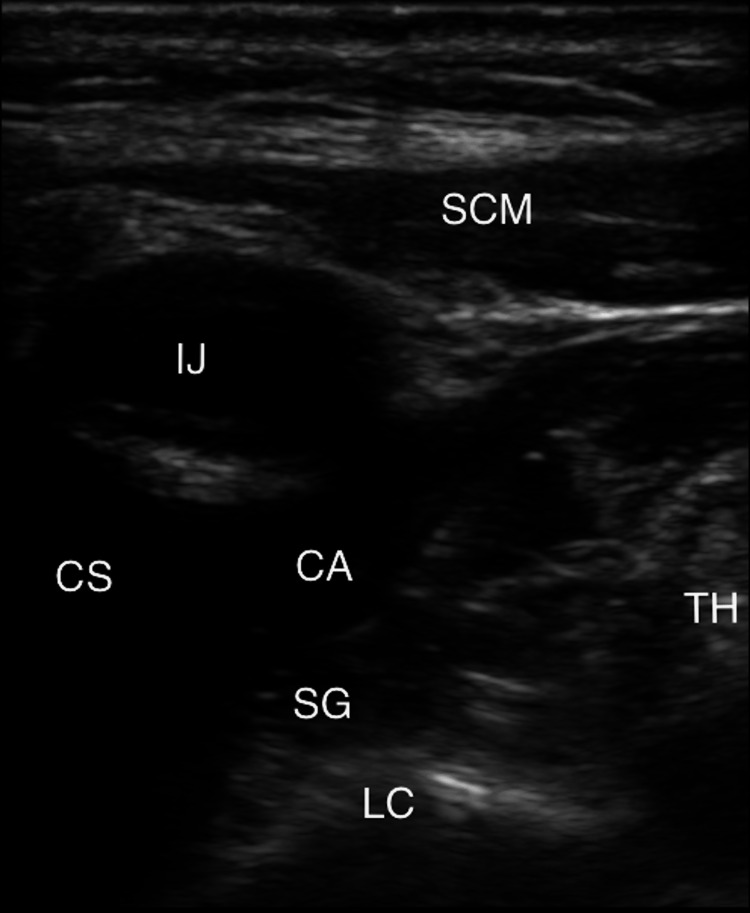
Right stellate ganglion block Ultrasound view of percutaneous blockade of the right stellate ganglion. Bilateral blocks were performed during the resuscitation. The C6 vertebral body likely lies deep (below) the visualized region on this ultrasound image frame. The injection is targeted slightly medial to lateral, beneath the carotid artery, and then lidocaine is injected to visualize the separation of the muscular and fascial layers to confirm correct placement. A bilateral blockade was performed. SCM - sternocleidomastoid muscle, IJ - internal jugular vein, CS - clavicular shadow, CA - carotid artery, SG - stellate ganglion, TH - thyroid tissue, LC - longus colli muscle.

During the following hour, the patient had an additional 60-second generalized electrographic seizure which then progressed to include 20-seconds of convulsions. Shortly after the onset of the convulsion, she developed a slow wide-complex ventricular tachycardia with cardiac arrest (Figure 2) which was observed by the authors. Cardiopulmonary resuscitation (CPR) and defibrillation (once), amiodarone was used to treat the arrhythmia, and escalating doses of IV lorazepam and ketamine were given to abort generalized seizure. Seizure activity was noted on EEG to continue after initiation of CPR, and she was later transitioned to IV midazolam. Given the refractory ventricular arrhythmia, bilateral stellate ganglion blocks [4-6] with bupivacaine (Figure 3) were employed to reduce the risk of recurrent arrhythmia. This resulted in expected sinus bradycardia and the need for inotrope administration. Subsequent metabolic and respiratory acidosis was corrected, and lacosamide was discontinued, given the arrhythmia. Femoral arterial and venous 5 Fr central line sheaths were placed in case of recurrent cardiac arrest to permit facile extracorporeal cardiopulmonary resuscitation (eCPR) [7-9] if it proved necessary, but she remained clinically stable and had normalization of her hemodynamics over the following 24 hours. The patient’s anesthetics were gradually weaned over the following 48 hours, and she was subsequently extubated. Unfortunately, she had a right hemiparesis and dense global aphasia, and subsequent computed tomography angiography (CTA) imaging of the brain showed a left middle cerebral artery ischemic stroke which was conservatively managed. 

## Discussion

Initial lacosamide blood levels from the day of admission (shortly after hospital arrival) resulted at 80.7 mcg/mL, over eight-fold the therapeutic upper limit [1]. This was obtained prior to additional dosing of lacosamide, but the results were not available until over 24 hours later after the conclusion of this episode. Her family suspected a suicide attempt given her recent behavior changes, paranoia, and depression history. The family retrieved her medication containers which, from pill counting, 26 (200 mg) lacosamide tablets were unaccounted for. Given her blood levels, the diagnostic conclusion was a probable intentional overdose of lacosamide, although her cardiovascular history may have been either a predisposing risk factor or an alternative explanation for this episode. The initial decision to use additional IV dosing of this medication in the emergency department, which had previously been successfully used to control her breakthrough seizures in prior presentations, was problematic in this circumstance, though her risk factors for intentional overdose were not known, and the patient was non-communicatory upon hospital arrival. Stellate ganglion blockade performed well at preventing further arrhythmia while serum levels of lacosamide ameliorated during the following days based on serial testing, and ultimately stabilized at trough levels of 6.6 mcg/mL on her originally prescribed home dosing. Due to medically necessary sedation and antiseizure medication, her ischemic stroke was not detected until she was outside the window for directed treatment. Given the lack of localizing features at the time of hospital arrival, the suspicion is that arrhythmia and/or cardiopulmonary resuscitation provoked a cardioembolic stroke though we cannot be certain. It is pertinent to note that stellate ganglion blockade has rarely been associated with stroke [10]; however, we suspect that the more pertinent risk factors, in this case, were arrhythmia and cardiac arrest. She was discharged to a nursing home due to disabling persistence of aphasia and hemiparesis; however, she ultimately improved enough from a motor perspective to return home with family supervision. Her aphasia remains severe. 

## Conclusions

This case is highly unusual but instructional in that it reviews both an uncommon but severe toxicity of lacosamide which can paradoxically cause both seizures and terminal cardiac arrhythmias. Acute care physicians should consider antiseizure medication overuse or intentional overdose in the differential diagnosis (especially with patients with well-controlled epilepsy), particularly if there is evidence of risk factors for intentional overdose. In the absence of corroborating history, this can be challenging as quantitative drug level assays generally do not result rapidly enough to change management. Stellate ganglion blockade and eCPR for the treatment and stabilization of refractory arrhythmia may be warranted in cases of severe lacosamide toxicity. 
